# *PepO* is a target of the two-component systems VicRK and CovR required for systemic virulence of *Streptococcus mutans*

**DOI:** 10.1080/21505594.2020.1767377

**Published:** 2020-05-19

**Authors:** Lívia A. Alves, Tridib Ganguly, Érika N. Harth-Chú, Jessica Kajfasz, José A. Lemos, Jacqueline Abranches, Renata O. Mattos-Graner

**Affiliations:** a Department of Oral Diagnosis, Piracicaba Dental School – State University of Campinas, Piracicaba, SP, Brazil; b Department of Oral Biology, University of Florida College of Dentistry, Gainesville, FL, USA

**Keywords:** *Streptococcus mutans*, systemic infections, two component system, peptidases, complement system, cardiovascular diseases

## Abstract

Streptococcus mutans

, a cariogenic species, is often associated with cardiovascular infections. Systemic virulence of specific *S. mutans* serotypes has been associated with the expression of the collagen- and laminin-binding protein Cnm, which is transcriptionally regulated by VicRK and CovR. In this study, we characterized a VicRK- and CovR-regulated gene, *pepO*, coding for a conserved endopeptidase. Transcriptional and protein analyses revealed that *pepO* is highly expressed in *S. mutans* strains resistant to complement immunity (blood isolates) compared to oral isolates. Gel mobility assay, transcriptional, and Western blot analyses revealed that *pepO* is repressed by VicR and induced by CovR. Deletion of *pepO* in the Cnm^+^ strain OMZ175 (OMZpepO) or in the Cnm^−^ UA159 (UApepO) led to an increased susceptibility to C3b deposition, and to low binding to complement proteins C1q and C4BP. Additionally, *pepO* mutants showed diminished *ex vivo* survival in human blood and impaired capacity to kill *G. mellonella* larvae. Inactivation of *cnm* in OMZ175 (OMZcnm) resulted in increased resistance to C3b deposition and unaltered blood survival, although both *pepO* and *cnm* mutants displayed attenuated virulence in *G. mellonella*. Unlike OMZcnm, OMZpepO could invade HCAEC endothelial cells. Supporting these phenotypes, recombinant proteins rPepO and rCnmA showed specific profiles of binding to C1q, C4BP, and to other plasma (plasminogen, fibronectin) and extracellular matrix proteins (type I collagen, laminin). Therefore this study identifies a novel VicRK/CovR-target required for immune evasion and host persistence, *pepO*, expanding the roles of VicRK and CovR in regulating *S. mutans* virulence.

## Introduction

*Streptococcus mutans* is a major bacterial species involved in the pathogenesis of dental caries [1–3], which is often associated with cardiovascular diseases, including infective endocarditis (IE) and apparently atheromatosis [4–8]. While the molecular functions involved in *S. mutans* cariogenicity are relatively well known, the mechanisms enabling *S. mutans* to cause extra-oral infections are poorly understood [1–3]. The four known *S. mutans* serotypes (*c, e, f,* and *k*) differ in detection rates in the oral cavity and extra-oral infections [9–12]. Serotype *c* is the most prevalent in the oral cavity worldwide, accounting for 53% to 80% of the strains [9–12]. Although less prevalent in the oral cavity, serotypes *e* (15–32% of the strains), *k* (1.4–35.2% of the strains), and *f* (4.7–8.5% of the strains) are detected at significant rates in extra-oral infections [9–12]. Although serotype distribution in cardiovascular tissues remains to be investigated in more detail, the largest PCR-based analyses of cardiovascular specimens revealed that most of the *S. mutans*-positive specimens harbor multiple serotypes [4]. Serotype *c* and *e* are the most prevalent in heart valves (30% and 48.5%, respectively) and atheromatous plaques (65.6% and 62.5%, respectively) [4]. Serotypes *f* and *k* show lower detection rates in heart valves (3.0% and 9.0%, respectively) and atheromatous plaques (9% and 24%, respectively), and were frequently associated with serotype *c* and/or *e* [4].

The collagen/laminin-binding protein Cnm has been previously implicated in *S. mutans* systemic virulence [13–17]. However, Cnm is mostly restricted to serotypes *f* and *k*; and is found in approximately 21% of the *S. mutans* isolates [12,14,18]. Cnm avidly binds to collagen and laminin, prevents platelet activation and is required for *S. mutans* cell invasion *in vitro*, colonization of the heart endothelium, and for virulence in the *Galleria mellonella* invertebrate model [15–17,19-21]. In addition, Cnm homologues bind to C1q, the first pattern recognition protein of the complement classical pathway, which might suggest a role for Cnm in complement evasion [20].

The ability to evade the complement system is a major virulence attribute in streptococcal pathogens [21–23]. In *S. mutans*, serotype *c*/*e* strains isolated from the bloodstream show increased resistance to complement immunity when compared to oral isolates [24,25]. Complement-resistance phenotypes of *S. mutans* are regulated by the two-component system (TCS) VicRK and CovR [24,25], an orphan response regulator orthologous to the TCS CovRS of *Streptococcus pyogenes* [26]. Both VicRK and CovR were shown to control the expression of *S. mutans* genes associated with the synthesis and binding to exopolysaccharides (*gtfB*/*C*/*D, gbpC, epsC*) [27]. However, contribution of these genes to complement evasion appears to be strain-specific [25]. Interestingly, VicRK and CovR also regulate *cnm* transcription in the serotype *f* strain OMZ175 [28], indicating a conserved role of these regulatory systems in controlling systemic virulence in *S. mutans*. Preliminary evidence indicated that the *pepO* gene, encoding for an endopeptidase O conserved among several streptococcal species [29–31], is another target of VicRK regulation in *S. mutans* [24]. In *S. pyogenes* and *S. pneumoniae*, PepO contributes to complement evasion by preventing activation of the classical pathway [32,33]. Moreover, *pepO* is regulated by the TCS CovRS in *S. pyogenes* [34].

In this study, we investigated *pepO* regulation by CovR and VicRK in *S. mutans*, and used Cnm^+^ (OMZ175) and Cnm^−^ (UA159) strains to probe the role of PepO in traits associated with immune evasion, host persistence, and virulence. Our findings expand the current knowledge about the scope of VicRK/CovR-regulated genes and identify PepO as an important virulence factor for *S. mutans* during systemic infection.

## Materials and methods

### Strains and culture conditions

Strains used in this study are described in Table 1 [15,18,24,28,35–37]. Strains were routinely grown in brain heart infusion (BHI) agar (BD Difco, USA) at 37°C in a 10% CO_2_ atmosphere. When needed, growth media were supplemented with appropriate antibiotics [erythromycin (10 μg/mL), spectinomycin (200 μg/mL), and/or kanamycin (1000 μg/mL)] (Merck Labs, Germany). Overnight cultures with adjusted absorbances were prepared in BHI, diluted 1:100 into fresh BHI or chemically defined medium (CDM) [38], and incubated for phenotypical analyses. *Escherichia coli* was grown in a 37°C shaker incubator in Luria-Bertani broth (BD Difco, USA) supplemented with ampicillin (100 µg/mL).

**Table 1.** Strains included in this study.

Strain	Serotype	Site of isolation and/or relevant characteristics	Source or reference
*Streptococcus mutans*			
UA159	*c*	Oral isolate, caries-affected child	ATCC
OMZ175	*f*	Dental plaque; *cnm+*	[15]
3SN1	*e*	Oral isolate; *cnm-*	[35]
8ID3	*c*	Oral isolate; *cnm-*	[35]
11SSST2	*c*	Oral isolate; *cnm-*	[35]
15VF2	*e*	Oral isolate; *cnm-*	[35]
SA13	*c*	Blood, bacteremia; *cnm-*	[18]
SA15	*e*	Blood, bacteremia; *cnm-*	[18]
SA16	*e*	Blood, infective endocarditis; *cnm-*	[18]
SA18	*c*	Blood, infective endocarditis; *cnm-*	[18]
UApepO	*c*	∆*pepO*::Erm^r^	[24]
UAcov	*c*	∆*covR*::Erm^r^	[36]
UAvic	*c*	∆*vicK*::Erm^r^	[37]
OMZpepO	*f*	∆*pepO*::Erm^r^	This study
OMZcnm	*f*	∆*cnm*::Kan^r^	[15]
OMZpepO/cnm	*f*	∆*pepO*::Erm^r^; ∆*cnm*::Kan^r^	This study
OMZcovR	*f*	∆*covR*::Erm^r^	[28]
OMZvicK	*f*	∆*vicK*::Erm^r^	[28]
UApepO+	*c*	pMC340B::*SMU_2036*; Kan^r^	This study
UAcov+	*c*	∆vicK::Ermr; pDL278::*SMU.1924*; Spec^r^	[36]
UAvic+	*c*	∆vicK::Ermr; pDL278::*SMU.1516*; Spec^r^	[37]
OMZpepO+	*f*	pMC340B::*SMU_2036*; Kan^r^	This study
OMZcovR+	*f*	pMC340B::*SMU_1924*; Kan^r^	[28]
OMZvick+	*F*	∆vicK::Ermr; pDL278::*SMU.1516*; Spec^r^	[28]
*Escherichia coli*			
DH10B-pETrepOSmu	-	pET16B::pepO	This study
BL21-pETrepOSmu	-	pET16B::pepO	This study

### Construction of isogenic mutants and complemented strains

Nonpolar inactivation of *pepO* in *S. mutans* strain OMZ175 was performed using a PCR-ligation strategy, as previously described. Briefly, the internal sequence of *pepO* (1,432 bp) was replaced by an erythromycin resistance cassette (Erm^r^) obtained from plasmid pVA838 [39]. The recombinant allele was transformed in OMZ175 or OMZcnm (to generate a double mutant OMZcnm/pepO) in the presence of the ComX-inducing peptide (XIP), as described elsewhere [40]. Transformants were selected on BHI agar containing erythromycin, and confirmed by PCR and DNA sequencing analysis. To construct a *pepO* complemented strain, full-length *pepO* was cloned into the integration vector pMC340B [41]. The resulting plasmid (pMCpepO) was transformed into the *pepO* mutant strain (OMZpepO) for integration at the *mtlA1* locus and selected on plates containing kanamycin. Plasmid integration was then confirmed by PCR and sequencing of the *mltA1* locus.

### Collection of serum and blood samples

Blood and serum samples were obtained from one healthy volunteer with normal ranges of C3, IgG, and IgM, and with reference profiles of complement-mediated opsonization, as revealed in previous comparisons with serum pools obtained from six subjects enrolled in a previous study [23]. Samples were obtained using standard protocols [25] under approval of the Ethical Committee of the Piracicaba Dental School, University of Campinas (UNICAMP) (proc. nº 153/2014), and of the National Commission on Ethics in Experimentation (CONEP), Brazil (CAAE: 83,140,418.0.0000.5418).

### S. mutans *binding to complement proteins*

Levels of C3b, C1q, and C4b-binding protein (C4BP) bound to the surface of serum-treated strains were determined as detailed elsewhere [25], with some modifications. Briefly, approximately 10^7^ CFU from mid-log phase cultures were harvested by centrifugation (10,000 x *g*, 4°C), washed twice with PBS (pH 7.4), and resuspended in 20 µl of 20% serum (diluted in PBS). After incubation at 37°C for 30 min, cells were washed twice with PBS-Tween 0.05% (PBST) and incubated with fluorescein isothiocyanate (FITC)-conjugated polyclonal goat anti-human C3 IgG antibody (1:300 in PBST; on ice for 40 min.) (ICN, USA), FITC-conjugated polyclonal goat anti-human C1q (1:300 in PBST; 37°C for 60 min.) (LSBio, USA) [42], or FITC-conjugated polyclonal rabbit anti-human C4BP (1:225 in PBST; 25°C for 60 min.) (LSBio, USA) [43]. Next, cells were washed twice with PBST and fixed in 3% paraformaldehyde in PBS for flow cytometry analyses using a FACSCalibur flow cytometer (BD Biosciences). A total of 25,000 bacterial cells were gated using forward and side scatter parameters. Results were expressed as the geometric mean fluorescence intensity (MFI) of C3b/C1q/C4BP-positive cells [25]. Control samples included cells treated only with PBS instead of serum. Heat-inactivated sera (56°C for 20 min) were used as negative controls in preliminary experiments for strain comparisons.

### RNA isolation, reverse transcription, and qPCR

RNA was purified from normalized number of bacterial cells at mid-log growth phase (A_550_ _nm_ 0.3) using RNeasy kit (Qiagen, USA) and treated with Turbo DNase (Ambion, USA), as previously described [25]. cDNA was obtained from 1 μg of total RNA using random primers [44] and SuperScript III (Life Technologies, USA), according to the manufacturer’s instruction. Quantitative PCR was performed in a StepOne™ Real-Time PCR System (Life Technologies, USA) with cDNA (10 ng), 10 μM of each primer, and 1× Power SYBR® Green PCR Master Mix (Lifetech, USA) in a total volume of 10 μl. Ten-fold serial dilutions of genomic DNA (300 to 0.003 ng) were used to generate standard curves for absolute quantification of RNA expression levels. The expression levels of the tested genes were normalized by the expression levels of the 16 S rRNA gene [27].

### Production of recombinant proteins and polyclonal antibody

Recombinant collagen-binding domain A of Cnm (rCnmA) comprised of 120 amino acids (position 175 to 294) of full-length Cnm protein (549 amino acids) was obtained as previously described [19]. To obtain His-tagged PepO recombinant protein (rPepO), a DNA fragment containing the entire PepO coding region (1896 bp) was amplified from UA159 genomic DNA using primers containing XhoI and SphI restriction sites at their 5ʹ ends (Table 2). The PCR-purified *pepO* fragment was then cloned into the expression vector pET-16B (Novagen, USA) to yield pET16B-rPepO. The resulting plasmid was transformed into *E. coli* BL21, and rPepO produced by growing cells to mid-log phase (A_600_ _nm_ ≈ 0.5) followed by induction with 1 mM isopropyl-β-D-thiogalactopyranoside (IPTG) (Sigma-Aldrich, USA) at 25°C for 18 h. rPepO was purified under native conditions using the Ni-NTA Protein Purification Kit (Qiagen, USA). Elution fractions were dialyzed in PBS (4°C for 18 h), and analyzed by SDS-PAGE and Western blot using an anti-6X-His tag antibody (Thermo Scientific Fisher, USA). The identity of purified rPepO was confirmed by mass spectrometry (ICBR Proteomics Mass Spectrometry, USA). Polyclonal antibodies against rPepO were produced using the standard 50 d Rabbit Protocol at Lampire Biological Laboratories (Ottsville, PA, USA).

**Table 2.** Oligonucleotides used in this study.

Primer	Sequence ^a^ (Forward/Reverse)	Product size (bp)	
Mutant construct			
P1 pepO P4 pepO	TACTATCGGCGCTAAGGT/ GATCAAAGGCAATTTACGG	2,384	
P1 *covR* P4 *covR*	CGT CTGCCAACTCATCCATAAC TCTATGAAACCTGTTGA/	2,038	
P1 *vicK* P4 *vicK*	TTACCAGATGCTTTTGTTGCT/ CTCTTGCCGTCTTTCATCAG	2,036	
C1 *covR*-SphI C2 *covR*-XhoI	CCTCTACCCAGCATGCCAATGGAAC/ GTCCAATTTCTCGAGTTATCGCGTG	1,039	
C1 *pepO*-SphI C2 *pepO-*XhoI	AAGAACATATGCATGCCCAATTCTGGG/ TCTAGTCAATGCTGGAGCGCTTGAA	2,169	
qPCR analysis			
*16 s-*RT-F *16 s-*RT-R	CGGCAAGCTAATCTCTGAAA/GCCCCTAAAAGGTTACCTCA	190	
*pepO*-RT-F *pepO*-RT-R	TACCCATAGCTTGAGGTGT/ ACACCAGAACTGCCTTTAG	253	
*vicK-*RT-F *vicK-*RT-R	CGGCGTGATGAATATGATGAA/GAGGTTAATGGTGTCCGCAGT	185	
*vicR-*RT-F *vicR-*RT-R	AGTGGCTGAGGAAAATGCTT/CATCACCTGACCTGTGTGTG	163	
EMSA			
SMU.2036-*pepO*-F SMU.2036-pepO-R	AGCTCCGCTTTATATTCCTG/ TGCTAGATACTAACCAATGCCT	321	
SMU.910-*gtfD*-F SMU.910-gtfD-R	TCTCTCCTGACCACTCCCTTA/TACCCAGTGCTTTTTAACCTTG	324	
SMU.1924-*covR*-F SMU.1924-covR-R	AGATGTCCTCTACCCATTGA/ CCTCATATCCTTCATGTTGTA	356	
Plasmid constructs			
rpepO_XhoI-F- pET16b rpepO_BamHI-R_pET16b	TGGAGACTCGAGATGGTACGTTTAC/ AAACTGGATCCCTACCAAATAATAACAC	1,919	

^a^Underlined sequences indicate restriction enzyme linkers.

### Protein extracts and Western blot analysis

PepO production by *S. mutans* strains was analyzed by Western blot using equal amounts of whole cell protein lysate (10 µg) obtained from mid-log phase cultures (A_550_ _nm_ = 0.3) grown in BHI, as previously described [27]. Analysis of the proportions of cell-associated and secreted PepO was performed in representative strains using cultures grown to stationary phase (A_550_ _nm_ = 1.0) in CDM, as described elsewhere [45]. Briefly, cells from 40 mL cultures were harvested by centrifugation (at 6,000 x *g*; 4°C; 4 min.), and the culture supernatants filtered through 0.22 µm pore size polyethersulfone membranes (Millipore, USA), neutralized and added of 10 µM of phenylmethylsulfonyl fluoride (PMSF) (Sigma-Aldrich, USA). Afterward, the culture supernatants were dialyzed against cold Tris-HCl (1.25 mM; pH 6.8) and 80-fold concentrated by freeze-drying. For the preparation of whole cell extracts, the harvested cells were washed twice with saline solution, suspended in 500 µl of MilliQ water, and mechanically disrupted in a Bead Beater (Biospec Products) with 0.16 g of 0.1 mm zirconia beads (2 cycles of 45 sec with 1 min rest on ice). The cell lysates were centrifuged (12,000 x *g*; 4°C; 1 min.) and the supernatants containing the whole protein extracts stored at −70°C. Protein concentration of whole cell and culture supernatant extracts was determined using a Bradford assay kit (BioRad, USA). For PepO detection, equal amounts of protein (10 µg) of cell extracts or concentrated culture supernatants were resolved in duplicate 10% SDS-PAGE gels, which were either stained with Coomassie blue (Sigma-Aldrich, USA) or transferred to polyvinylidene fluoride (PVDF) membranes for Western blot analysis. PepO was then probed using rabbit anti-rPepO antiserum (1:1,000), followed by incubation with goat anti-rabbit IgG antibody conjugated with horseradish peroxidase (1:10,000). Antibody reactions were detected using the Pierce ECL system (Pierce). Probed PepO was quantified by densitometry, using ImageJ Processing Analysis software in Java (NIH, http://rsbweb.nih.gov/ij/index.html), and expressed as arbitrary units. Densitometric measures of PepO bands were performed within a linear range of detection, as determined using a standard curve of rPepO (0.01 to 1 µg/mL).

### Binding of rPepO or rCnmA to host glycoproteins

Binding of rPepO and rCnmA to immobilized human glycoproteins was determined as previously described [29] with some modifications. Briefly, wells of 96-well microtiter plates (Greiner Bio-One) were coated (18 h at 4°C) with a solution of 5 μg/mL (in 75 mM sodium carbonate buffer pH 9.6) of plasma (plasminogen, fibronectin, fibrinogen) (Sigma-Aldrich, USA), type I collagen or laminin (both from human fibroblast, Sigma-Aldrich, USA), or complement proteins [C1q (Calbiochem, USA) and C4BP (Athens Research & Technology, USA)]. Wells coated with 1% BSA were used as control. Plates were then incubated (25°C for 2 h) with blocking solution (50 mM Tris-HCl pH 8, 150 mM NaCl, 0.1% Tween 20, 3% fish gelatin), and rPepO (10 to 50 µg/mL), or rCnmA (0.125 to 2 µg/mL) diluted in binding buffer (50 mM HEPES pH 7.4, 150 mM NaCl, 2 mM CaCl2, and 50 μg/mL BSA). After incubation (2 h at 37°C), wells were washed with PBST, and bound rPepO or rCnmA probed with rabbit rPepO (1:1,000) or rCnmA (1:3,000) antiserum (2 h at 37°C), followed by incubation (1 h at 37°C) with goat IgG anti-rabbit IgG conjugated with peroxidase (1:10,000) (Sigma-Aldrich, USA). Plates were washed three times with PBST and incubated with 3,3′,5,5′-tetramethylbenzidine (TMB) peroxidase substrate (100 µl) for up to 10 min for antibody detection. The absorbances (A_450_ _nm_) were then determined using a microplate reader (VersaMax) and expressed as measures of bound rPepO or rCnmA.

### Galleria mellonella *infection*

The *G. mellonella* infection model was used to compare the virulence potential of *S. mutans* strains as previously described [15,19], with some modifications. Briefly, strains were grown in BHI (37°C for 18 h), washed, and resuspended in sterile saline to 1 × 10^8^ CFU/mL. Aliquots of 5 µl of these suspensions were then injected into the hemocoel at the last left proleg of each larva. Suspensions of heat-inactivated strains (80°C for 30 min) or saline were used as controls in each experiment. To further assess the contribution of PepO to *S. mutans* virulence, strain UA159 was pre-incubated (30 min at 37°C) with rabbit anti-rPepO antiserum (1:10), washed and resuspended in sterile saline before injection. Controls included injection of anti-rPepO alone and UA159 pre-incubated with pre-immune serum. After injection, larvae were kept in the dark at 37°C, and their survival over time recorded. Three independent experiments were performed, each using three individual biological replicates.

### Survival in human blood

Survival of *S. mutans* in human blood *ex vivo* was analyzed as previously described [25]. Briefly, bacteria were grown in BHI to A_550_ _nm_ 0.3, harvested by centrifugation (11,000 x *g*, 2 min.), washed twice in PBS, and resuspended in 1 mL of fresh whole human blood. Blood suspensions were then incubated (37°C in a 10% CO_2_ atmosphere with gentle agitation), and aliquots collected at selected intervals for determination of bacterial counts by plating serially diluted aliquots on BHI agar. Aliquots collected just after the bacteria were suspended in blood were analyzed to confirm that blood-mediated aggregation did not influence bacterial counts. Three independent experiments were performed in duplicate.

### Invasion of human coronary artery endothelial cells (HCAEC)

HCAEC invasion by *S. mutans* was assessed as previously described [15]. Briefly, primary HCAEC (Lonza, USA) were cultured in endothelial cell basal medium 2 (EBM-2; Lonza) supplemented with 2% fetal bovine serum (FBS) (37°C, 5% CO2, humidified atmosphere). Cells were then harvested by trypsinization, washed in EBM-2, and resuspended in the same medium to 10^5^ cells/mL. Aliquots of 1 mL of these suspensions were seeded in 24-well flat-bottomed tissue culture plates containing EBM-2 supplemented with endothelial growth factors (Lonza SingelQuots) and 10 µg/mL gentamicin. Then, cells were washed with fresh pre-warmed EBM-2 medium without antibiotics, and co-cultured for 2 h with 10^7^ CFU of *S. mutans* (MOI 100). Extracellular bacteria were removed by washing the wells twice with pre-warm PBS and killed with EBM-2 medium containing 300 μg/mL gentamicin and 50 μg/mL penicillin G to kill extracellular bacteria for 2 h. HCAEC cells were washed three times with pre-warm PBS and lysed by incubation in 1 mL of sterile cold water for 20 min. Lysed HCAEC were plated on Tryptic soy agar (TSA) and incubated at 37°C for 48 h for CFU quantification. The numbers of intracellular bacteria were then expressed as the percentage of invasion for each strain in relation to the initial inoculum.

### Electrophoretic mobility shift assays (EMSA)

EMSAs were performed using recombinant CovR (rCovR) and VicR (rVicR) obtained in a previous study [27]. Amplicons of the promoter regions of *pepO* (500 pb upstream of pepO start codon) were obtained using specific primers (Table 2). As controls, amplicons of the promoter regions of *covR* (356 bp; negative control of VicR binding) and *gtfD* (324 bp; negative control of CovR binding) were also obtained using the primers described in Table 2. The PCR products were purified (QIAquick PCR Purification Kit, Qiagen) and labeled with biotin, using the Biotin 3ʹ End DNA Labeling Kit (Thermo Scientific Fisher, USA). Binding reactions of labeled DNA (≈ 20 fmoles) with rCovR or rVicR were carried out in volumes of 20 µL containing 1X Binding Buffer [100 mM Tris, 500 KCl, 10 mM DTT; pH 7.5], poly L-lysine (50 ng/μl), and unspecific competitor poly d(I-C). Samples were incubated for 45 min at 25°C, and DNA-protein complexes separated in non-denaturing 6% acrylamide gels in 0.5 X TBE buffer (pH 8.0). Protein-DNA complexes were transferred to positively charged nylon membranes (Thermo Scientific Fisher, USA), probed with Stabilized Streptavidin Horseradish Peroxidase conjugate (Thermo Scientific Fisher, USA) and detected using the LightShift Chemiluminescent EMSA system (Thermo Scientific Fisher, USA). As controls for the specificity of protein binding, a 200-fold excess of unlabeled test fragment (*pepO* fragment) and unlabeled negative control DNA fragments (*gtfD* or *covR* fragments for rCovR and rVicR, respectively) were incubated with rCovR or rVicR in each reaction.

### Data analyses

Densitometric measures of PepO probed in Western blot analysis and transcript levels of the studied genes were compared between groups using Mann–Whitney U-test. Ability of bacteria to bind to complement proteins (MFI values of C3b/C1q/C4BP) and HCAEC invasion were compared between strains using nonparametric Kruskal–Wallis *post hoc* Dunns’ test. Bacterial counts determined in the human blood survival assay were compared between strains at each time point, using Kruskal–Wallis *post hoc* Dunns’ test, with correction for repeated measures. Kaplan-Meier survival curves generated in the *G. mellonella* killing assays were compared using log-rank test. ANOVA with *post hoc* Dunnett’s was used to test differences in *S. mutans* or rPepO/rCnmA binding to plasma or host glycoproteins. Differences were considered significant when p values were less than 0.05.

## Results

### Expression levels of pepO are higher in strains resistant to complement deposition compared to nonresistant strains

**Figure 1.**
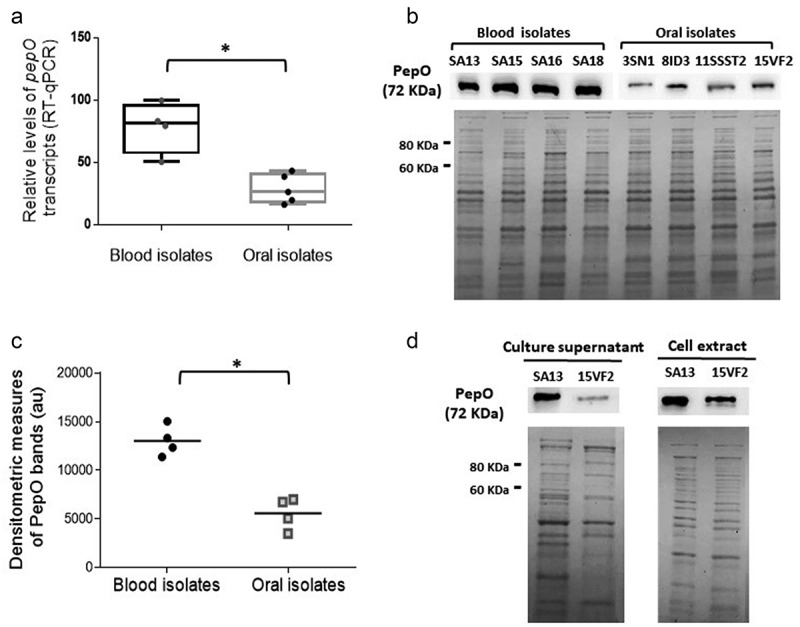
PepO expression in *S. mutans* strains. (a) Box plot comparisons of relative levels of *pepO* transcripts in serotype *c*/*e* strains with reduced C3b-binding isolated from the bloodstream (SA13, SA15, SA16, SA18) and oral stains with significant binding to C3b (UA159, 3SN1, 8ID3, 11SSST2, 15VF2). Levels of *pepO* transcripts were normalized by the respective levels of 16 S rRNA gene transcripts, as determined by RT-qPCR in samples with equivalent numbers of bacterial cells at A_550_ _nm_ 0.3. (b) Western blot analysis of PepO production. Protein extracts (10 µg) obtained from each strain (at A_550_ _nm_ 0.3) were resolved in 10% SDS-PAGE gels, which were either transferred to PVDF membranes to probe PepO with anti-rPepO antibodies (upper panel) or stained with Coomassie blue (lower panel) to monitor protein integrity. Images are representative of three independent experiments. (c) Comparisons of densitometric arbitrary units (au) of PepO detected in the Western blot analysis. Values represent mean densitometric measures of PepO obtained in three independent cultures. Asterisks indicate significant differences between groups (Mann–Whitney U-test; *p* < 0.05). (d) Western blot analysis of PepO in culture supernatants and cell factions in representative strains (SA13 and 15VF2). Western blot assays (upper panels) were performed with equivalent amounts of protein (10 µg) from culture supernatants or from bacterial cells, which were collected from CDM cultures (A_550_ _nm_ 1.0). Protein integrity was monitored in Coomassie blue stained SDS-PAGE gels (lower panel).

C3b is the major opsonin of the complement, which plays central downstream functions on the amplification of complement responses [46]. Previously, we reported that strains isolated from the bloodstream show reduced binding to C3b, when compared to oral isolates [25]. Here, we compared the transcriptional profile of *pepO* between sub-sets of these previously studied strains (four blood isolates with the lowest binding to C3b and four oral isolates with higher C3b binding). As shown in Figure 1(a), RT-qPCR analyses revealed increased *pepO* transcript levels in low C3b-binding blood strains (SA13, SA15, SA16, SA18) compared to oral strains (UA159, 3SN1, 8ID3, 11SSST2, 15VF2) (p < 0.05). These transcriptional profiles were consistent with the levels of PepO detected in whole cell extracts (Figure 1(b,c)). Because the *S. pneumoniae* PepO homologue (66% amino acid identity with *S. mutans* PepO) was reported to be secreted [29], we also assessed levels of secreted PepO among strains. As shown in Figure 1(d), PepO was detected in the culture supernatants of both representative strains (SA13 and 15VF2) at levels comparable to those found in cell extracts. These results indicate that strains resistant to C3b deposition produce higher levels of PepO when compared to strains with increased binding to C3b.

### PepO is directly regulated by CovR and by VicRK

**Figure 2.**
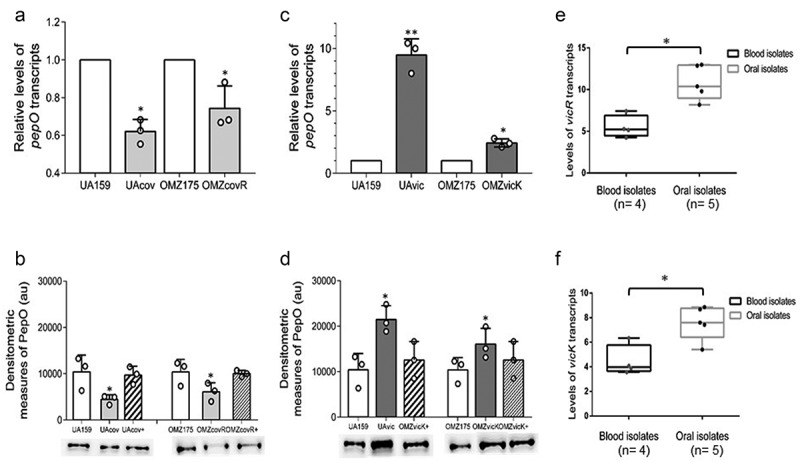
Regulation of *pepO* by the TCS VicRK and CovR orphan regulator in *S. mutans* strains. (a,c) Comparisons of *pepO* transcript levels between *covR* (UAcov and OMZcov) or *vicK* (UAvic and OMZvic) isogenic mutants with the respective parent strains (UA159 and OMZ175). Transcript levels were determined by RT-qPCR with specific primers in cells at A_550_ _nm_ 0.3. PepO transcript levels were normalized by 16 S rRNA gene transcripts. (b,d) Comparisons of the amounts of PepO produced by mutant, parent, and complemented (+) strains. Relative amounts of PepO were determined in proteins extracts (10 µg) obtained from cells at A_550_ _nm_ 0.3 by densitometry of PepO bands probed with anti-rPepO antibodies in Western blot assays. Columns represent means of densitometric measures of blots obtained in three independent experiments (individual values represented by circles); bars indicate standard deviations. Representative blots are shown in lower panels. Asterisks indicate significant differences in relation to the respective parent strain (Kruskal–Wallis *post hoc* Dunn’s test; *p* < 0.05). (e,f) Comparisons of transcriptional activities of *vicR* and *vicK* (encoding for the VicRK TCS) between blood and oral isolates. Transcript levels of *vicR* and *vicK* were determined by RT-qPCR with specific primers and normalized by 16 S rRNA gene transcripts in cells at A_550_ _nm_ 0.3. Asterisks indicate significant differences between groups (Mann–Whitney U-test; *p* < 0.05).

Because CovR regulates genes for complement evasion in *S. mutans* [25], we further investigated CovR role in *pepO* regulation. We analyzed the effects of *covR* inactivation on *pepO* transcriptional activities in two strain backgrounds, the Cnm- serotype *c* UA159 strain and the Cnm+ serotype *f* strain OMZ175. We found that *pepO* mRNA levels were 46% and 26% lower in the *covR* mutants of UA159 (UAcov) and OMZ175 (OMZcovR), when compared to their respective parent strains (ANOVA, p < 0.05) (Figure 2(a)). The reduced transcriptional levels of *pepO* in the *covR* mutants were also consistent with the reduced levels of PepO produced in these strains. Genetic complementation of the *covR* mutants restored PepO production to similar levels of the respective parent strains (Figure 2(b)). These results indicate that CovR induces *pepO* transcription. Because the studied blood strains with increased expression levels of *pepO* (Figure 1(a–c)) were previously shown to have reduced activity of *covR* [25], we addressed if *pepO* was also regulated by VicRK. There is previous evidence that *pepO* activity may be controlled by the VicRK system in the *S. mutans* strain UA159 [24]. Because *vicR* is essential for *S. mutans* viability [27,47], transcript levels of *pepO* were compared between *vicK* mutants obtained in UA159 (UAvic) and in OMZ175 (OMZvic) with the respective parent strains at A_550_ _nm_ 0.3. As shown in Figure 2(c), *pepO* transcript levels were approximately 9.5-fold higher in UAvic and 2.4-fold higher in OMZvicK (p < 0.05), indicating that VicRK negatively regulates *pepO* transcription. Transcriptional changes of *pepO* in the *vicK* mutants were consistent with increased amounts of PepO produced by these strains (Figure 2(d)), which were restored to parent levels in the respective complemented strains. Finally, we investigated transcriptional activities of *vicR and vicK* in blood and oral isolates, which differ in *pepO* expression levels (Figure 1). As shown in Figure 2(e,f), blood strains with increased *pepO* expression levels showed reduced levels of *vicR* and *vicK* transcripts when compared to oral strains, further indicating that VicRK negatively regulates *pepO* in *S. mutans*.

**Figure 3.**
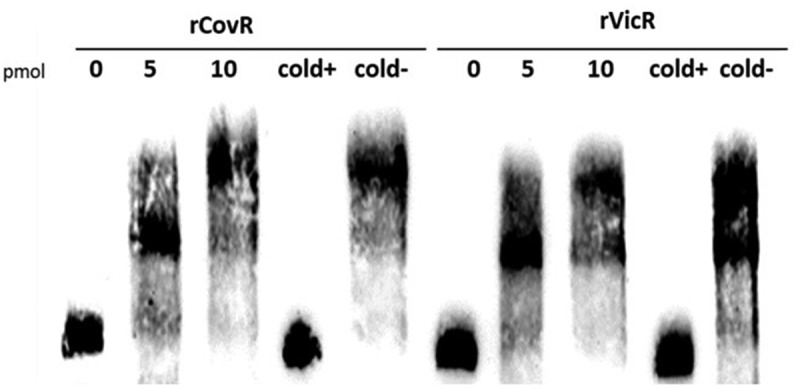
EMSA analysis of rCovR and rVicR binding to the promoter region of *pepO*. Specificity of binding was confirmed in competitive assays with excess of unlabeled test *pepO* DNA fragment (cold+) and excess of unlabeled negative control DNA fragments (*gtfD* and *covR* fragments for rCovR and rVicR binding, respectively; cold-). Image is representative of three independent experiments.

We next investigated whether the regulation of *pepO* by CovR and VicR is direct or indirect. Comparisons of *pepO* promoter regions in UA159 and OMZ175 revealed that these sequences (321 bp in length: 82 to 403 bp upstream to *pepO* translation start sites) are identical. In addition, besides two putative VicR-binding boxes in the *pepO* promoter region that have been previously reported, we also found a potential AT-rich CovR-binding site (AAATTTTTAcgAAgAA; mismatches in lower cases) located 350 bp upstream of the *pepO* translation start site. By using gel mobility shift assays, we investigated the ability of recombinant rVicR and rCovR proteins to bind to the 321 bp fragment amplified from *pepO* promoter region. As shown in Figure 3, rCovR or rVicR specifically retarded migration of the *pepO* promoter fragment amplified from UA159. Thus, our findings revealed that *pepO* transcription is directly regulated by both CovR and VicR, which respectively act as positive and negative regulators of *pepO* in different *S. mutans* serotypes.

### *Deletion of* pepO *increases C3b deposition and impairs binding to C1q and C4BP in Cnm^+^ and Cnm^−^ strains*

**Figure 4.**
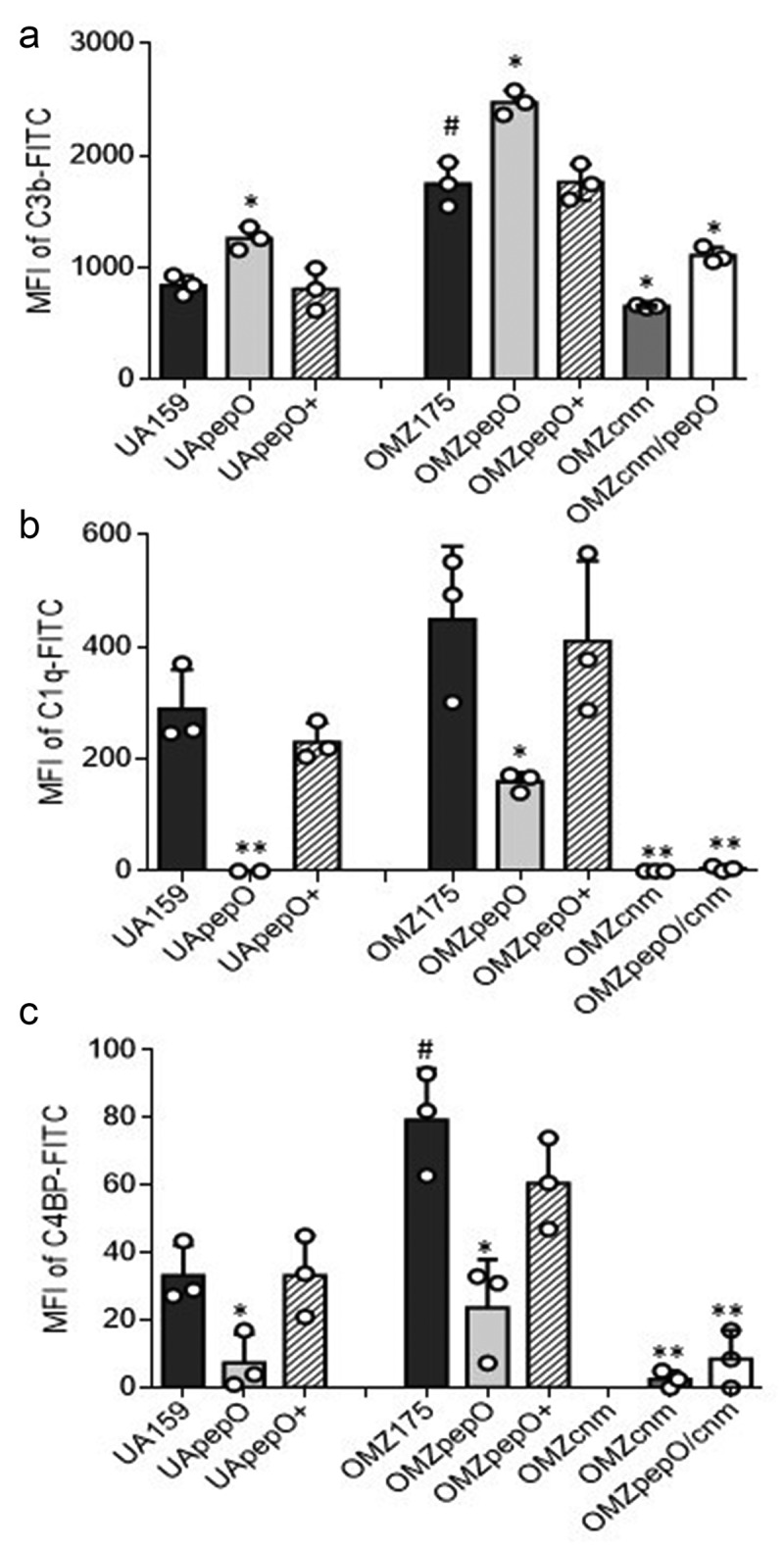
Effects of *pepO* and/or *cnm* inactivation on *S. mutans* interactions with complement proteins. Isogenic *pepO* (UApepO and OMZpepO) and *cnm* (OMZcnm) single mutants, as well as the OMZ175 double mutant (OMZcnm/pepO) were compared to the respective parent (UA159 or OMZ175) and complemented (+) strains. Strains were treated with 20% human serum and surface-bound C3b (a), C1q (b), or C4BP (c) probed with specific antibodies conjugated with FITC. Levels of surface-bound complement proteins were analyzed by flow cytometry and expressed as the geometric mean fluorescence intensity (MFI) of positive cells. Columns represent means of three independent experiments (individual values are represented with circles); bars represent standard deviations. Asterisks indicate significant differences in relation to the respective parent strain; hashtags indicate significant difference in relation to UA159 (Kruskal–Wallis with Dunn’s *post hoc* test; ^#,^ * *p* < 0.05; ** *p* < 0.01).

Previously, we reported that deletion of *covR* or *vicK* in the UA159 strain strongly reduces binding to C3b [24,25]. In this same strain, we also observed that deletion of *pepO* increased C3b deposition [24], suggesting a role for PepO in complement evasion. However, the specific interaction of *S. mutans* PepO with complement proteins was not investigated at that time. Notably, there is evidence that the *S. pneumoniae* PepO interferes with C3b deposition by sequestering C1q, the first pattern recognition protein of the classical complement pathway [32]. In addition, Cnm has been shown to interfere with complement activation, by also binding to C1q [20]. Here, we investigated whether deletion of *pepO* and *cnm* individually or in combination affected C3b deposition and C1q binding. Deletion of *pepO* significantly increased susceptibility to C3b deposition after treatment with human serum in both UA159 and OMZ175 background strains (49.6% and 41.7% increases, respectively) (Figure 4(a)), a phenotype that was restored in the complemented strains UApepO+ and OMZpepO+ (Figure 4(a)). On the other hand, the OMZ175cnm strain showed a 73.5% reduction in C3b deposition (Figure 4(a)), while inactivation of *pepO* in the OMZ175cnm strain increased C3b deposition, without fully increasing C3b binding to the OMZ175 levels (Figure 4(a)).

Deletion of *pepO* in OMZ175 impaired binding to C1q and virtually abolished C1q binding in the UA159 background strain; both phenotypes were restored in the complemented strains (Figure 4(b)). Binding to C1q was also abolished in the OMZcnm and OMZcnm/pepO strains (Figure 4(b)). In addition to binding to C1q, the *S. pneumoniae* PepO was also shown to affect C3b deposition by binding to the fluid phase complement downregulator C4BP [32]. Binding to C4BP mirrored the C1q binding profile in all strains tested (Figure 4(c)), indicating that PepO and Cnm also mediate binding to C4BP. Notably, OMZ175 showed increased binding to C1q and C4BP when compared to UA159, whereas deletion of *cnm* dramatically impaired binding to these complement proteins, indicating an important role of Cnm in the interaction of *S. mutans* with these complement proteins (Figure 4(b-c)).

### PepO and Cnm bind different panels of human glycoproteins involved in complement activity and tissue integrity

**Figure 5.**
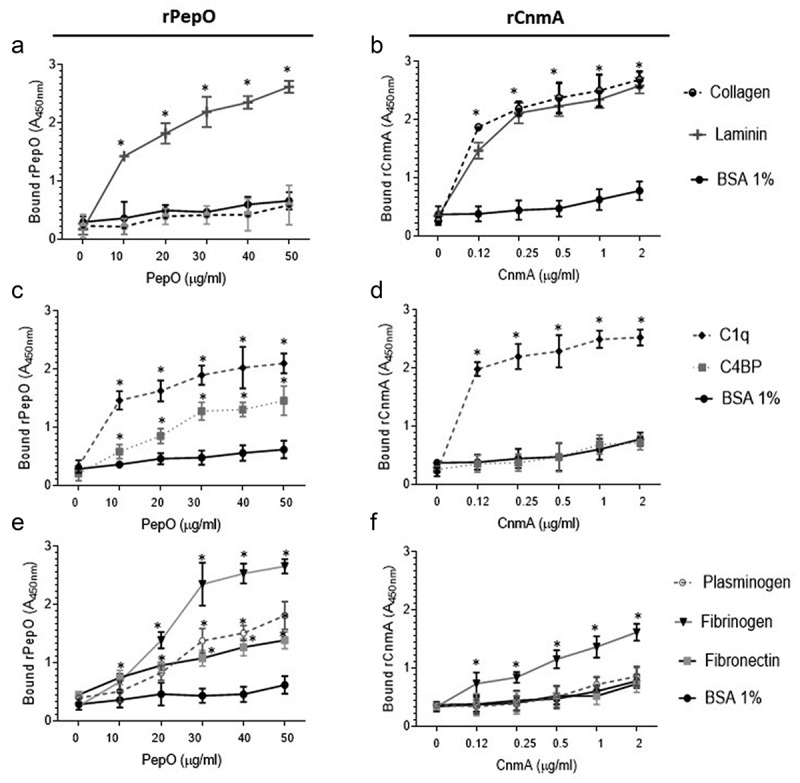
Binding of rPepO and rCnmA to human glycoproteins of the extracellular matrix, complement system, or plasma. Microtiter plates were coated with 5 µg/mL of each human glycoprotein and incubated with increasing amounts of rPepO (0–50 µg/mL) or rCnmA (0–2 µg/mL). BSA at 1% was used as control. Binding was detected using specific anti-PepO and anti-CnmA polyclonal antibodies. Data represent means of three independent experiments performed in triplicate. Bars indicate standard deviations. Asterisks indicate significant differences in relation to the respective control sample (ANOVA with *post hoc* Dunnett’s test; *p < 0.01).

Next, we compared binding affinities of rPepO and rCnm to human glycoproteins involved in complement activation (C1q, C4BP, plasminogen, fibrinogen) and tissue integrity (type I collagen and laminin). Here, we used rCnmA, which comprises the entire N-terminal collagen-binding domain A (288 amino acid length) of Cnm [19], and the full-length PepO. While rPepO did not bind to collagen, it bound strongly to laminin (Figure 5(a)), an extracellular matrix protein typically found in basement membranes associated with endothelial cells, aortic and heart muscle cells [48]. As shown previously [19], rCnmA showed strong binding to both collagen and laminin (Figure 5(b)). As expected, rPepO was also able to bind to C1q and to C4BP (Figure 5(c)). On the other hand, rCnmA bound strongly to C1q but failed to bind to C4BP (Figure 5(d)). rPepO strongly interacted with fibrinogen, and moderately interacted with plasminogen or fibronectin (Figure 5(e)). Finally, rCnmA bound to fibrinogen but not to plasminogen or fibronectin (figure 5(f)).

### PepO contributes to persistence in human blood and to HCAEC invasion

**Figure 6.**
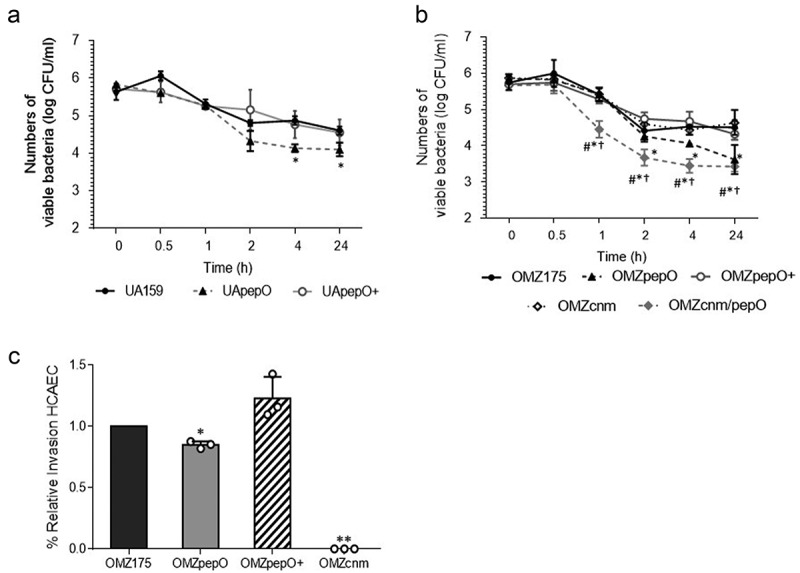
*Ex vivo* persistence in human blood and invasion to Human Coronary Artery Endothelial Cells (HCAEC) by *S. mutans* strains. (a,b) Equal numbers of bacterial cells were suspended into fresh human blood, and bacterial counts (log CFU/mL) determined at each time point of incubation (37°C). (a) Comparisons of cell counts of *pepO* mutant (UApepO), with parent (UA159) and complemented (+) strains. (B) Comparisons of cell counts of the single (OMZpepO and OMZcnm) or double (OMZcnm/pepO) mutants with parent (OMZ175) and complemented (+) strains. Data represent means of three independent experiments performed in triplicate. Bars indicate standard deviations. Asterisks indicate significant differences in relation to parent strain at each time point; symbols indicate significant differences between the double mutant OMZcnm/pepO and OMZpepO (#) or OMZcnm (†) (Kruskal–Wallis *post hoc* Dunn’s test using correction for repeated measures; *p* < 0.05). (C) Invasion of HCAEC by *S. mutans* strains. HCAEC were incubated with equal numbers of OMZ175, OMZcnm OMZpepO or OMZpepO+ complemented strain. After removal of extracellular bacteria by treatment with gentamicin and penicillin, *S. mutans* strains were recovered from intracellular compartment. The numbers of intracellular bacteria (CFU) were relative to parent strain OMZ175. Columns represent means of three independent experiments performed in duplicate (individual values are represented with circles). Bars represent standard deviations. Asterisks indicate significant differences in relation to OMZ175 (Mann–Whitney test; * *p* < 0.05; ** *p* < 0.01).

The complement system plays multiple functions for clearance of pathogens from the bloodstream [49,50]. Thus, we sought to determine the importance of PepO for *S. mutans* persistence in human blood *ex vivo*. When compared to the parent strain, deletion of *pepO* in UA159 caused small albeit significant reductions in survival after 4 and 24 h of incubation (17% and 12% reduction, respectively), a phenotype that was restored in the complemented strain (Figure 6(a)). In the OMZ175 background, the *pepO* mutant also showed notable reductions in survival after 4 h of incubation, which remained lower for 24 h (reductions ranging from 11% to 24%) (Figure 6(b)). As previously observed, inactivation of *cnm* in OMZ175 did not affect survival in blood. However, survival was further reduced in the OMZ175cnm/pepO double mutant when compared to the OMZpepO (Figure 6(b)).

It has been demonstrated that Cnm mediates the ability of *S. mutans* to invade endothelial and epithelial cells [15,51]. To address whether *pepO* plays a contributing role in the ability of OMZ175 to invade endothelial cells, antibiotic protection assays were performed. When compared to the parent OMZ175 strain, the OMZpepO mutant displayed a small (approximately 20%) reduction in its capacity to invade HCAEC, a phenotype that was restored in the *pepO*-complemented strain (Figure 6(c)). As expected, the invasive phenotype was abolished in the *cnm* mutant OMZcnm (Figure 6(c)). We conclude that PepO has a modest contribution to HCAEC invasion *in vitro*.

### *PepO is required for virulence in* Galleria mellonella

**Figure 7.**
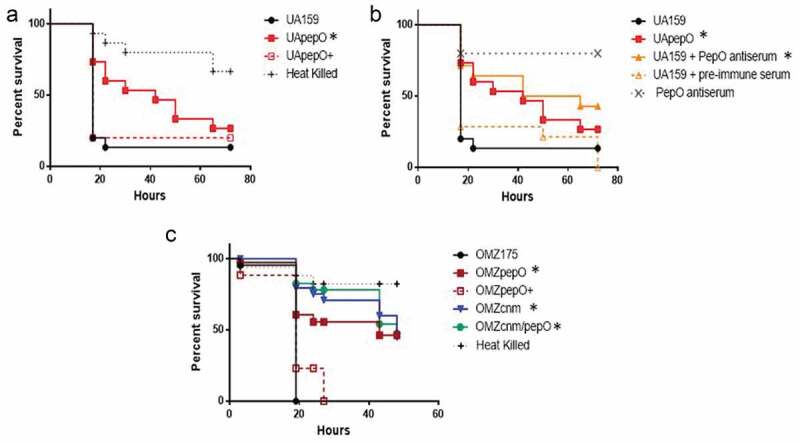
Killing of *Galleria mellonella* larvae infected with *S. mutans* strains. (a) Percents of larvae survival were compared between *pepO* mutant (UApepO) and the parent (UA159) or complemented (UApepO+) strains. (b) Percent survival of larvae, infected with UA159 treated or not with PepO antiserum to block PepO or with UApepO single mutant. UA159 treated with pre-immune serum was also used as control. (c) Comparisons of percent survival of larvae infected with single (OMZcnm or OMZpepO) or double mutant (OMZcnm/pepO) strains with parent (OMZ175) and complemented (OMZpepO+) strains. Heat killed bacteria and saline were used as negative controls in all the experiments. The results are representative of three independent experiments. Asterisks indicate significant differences in relation to parent strain (Kaplan–Meier curves compared using log rank test; *p < 0.01).

The immune system *of G. mellonella* includes complement-like proteins, opsonins, phagocytic cells (hemocytes), and anti-microbial peptides, thereby offering relevant parallels to the human innate immune system [52]. Using this model, we found that deletion of *pepO* in the UA159 background strain significantly reduced its capacity to kill *G. mellonella* larvae, a phenotype that was restored in the complemented UApepO+ strain (Figure 7(a)). Consistent with this finding, pre-incubation of UA159 with rPepO anti-serum protected *G. mellonella* against killing by the parent UA159 strain, whereas incubation with pre-immune serum conferred no protection (Figure 7(b)). Previously, we showed that Cnm is a major virulence factor for *S. mutans* in the *G. mellonella* model [15]; therefore, we also examined the relevance of PepO in *S. mutans*-mediated *G. mellonella* killing in the Cnm+ OMZ175 strain. Remarkably, inactivation of *pepO* significantly reduced OMZ175 virulence (Figure 7(c)). However, inactivation of both genes (OMZcnm/pepO strain) did not further attenuate virulence when compared to the single mutant strains (Figure 7(c)).

## Discussion

Although VicRK and CovR/CovRS have species-specific functions in transcriptional gene regulation among the streptococci [26], these regulatory systems share a major function in regulating genes involved in bacterial responses to envelope stresses and evasion to host immunity, including the complement system [25,27,47,53–56]. In *S. mutans*, differential expression of VicRK/CovR–targeted genes (*gtfB, gtfC, gbpB, gbpC, epsC*) within clinical strains significantly influences expression of virulence-associated phenotypes [25,35,45]. In this study, we identified *pepO* as a novel target of VicRK and CovR dual regulation. In addition, we showed that PepO is required for complement immunity evasion and virulence in a *G. mellonella* infection model. Thus, this study describes a novel virulence factor of *S. mutans* and expands the roles of VicRK and CovR as regulators of systemic virulence.

PepO proteins are conserved endopeptidases with M13 metallopeptidase domains, found in several streptococcal pathogens including *S. pneumoniae, S. pyogenes, S. suis* as well as oral streptococci such as *S. gordonii, S. sanguinis,* and *S. oralis* [29–31]. Although PepO does not possess signal peptides or cell wall anchor domains, PepO is detected in cell fractions as well as in culture supernatants of *S. pyogenes* and *S. pneumoniae* in a growth phase-dependent fashion [29,33,57]. In this study, we also show that PepO produced by *S. mutans* strains is present either in cell fractions or in culture supernatants. Although the mechanisms of PepO secretion remain to be elucidated, most functions reported in this study as well as in studies with other streptococcal species are compatible with the notion that PepO has important extracellular functions. These functions include PepO binding to complement proteins (C1q and/or C4BP) and to host tissue components, including fibrinogen, plasminogen, and fibronectin. Intracellular functions for PepO in promoting virulence were also reported in *S. pyogenes*, including cytoplasmic PepO peptidase activities on pheromone quorum-sensing peptides (SHP; short hydrophobic peptides) [34] and a role in transcriptional regulation of *speB* virulence gene [57]. More recently, new evidence emerged that *S. mutans* PepO might degrade XIP intracellularly, thereby affecting competence induction [58]. Our findings that PepO is directly regulated by VicR might support the hypothesis that PepO exposure to the cell surface involves the expression of murein hydrolases, which are typically regulated by VicRK during growth in *S. mutans* [27,37,47] and other Gram-positive bacteria [26,59]. We have previously observed increased expression of murein hydrolase genes (*smaA* and *smu.2146 c*) in the *vicK* mutant (UAvic) [24], in which *pepO* is up-regulated. To date, there is evidence that PepO of *S. pyogenes* is directly repressed by CovR [34,57]. However, our results revealed that the *S. mutans* CovR orphan regulator works as an inducer of *pepO*. CovR also represses *cnm in* OMZ175 [28], whereas both genes *pepO* (Figures 2 and 3) and *cnm* [28] are repressed by VicRK. Thus, VicRK and CovR work in concert to finetune the expression of genes involved in *S. mutans* systemic virulence.

In *S. pyogenes*, PepO binds to C1q affecting its interaction with IgG, promotes resistance to serum-mediated lysis, and contributes to virulence in a skin infection model [33]. Additionally, the *S. pneumoniae* PepO is required for complement evasion by recruiting fluid-phase components C1q and C4BP, a major down-regulator of the lectin and classical pathways of complement [29,32]. The classical pathway is crucial for complement activation on *S. mutans* strains [25]. Besides PepO, Cnm-like proteins expressed by different Gram-positive bacteria bind to C1q, reducing activation of the classical pathway [20]. In this study, we found that the deletion of *pepO* impairs bacterial binding to C1q and to C4BP, and consistently increases C3b deposition in *S. mutans* strains. Unexpectedly, deletion of *cnm* in OMZ175 also impairs binding to C1q and C4BP but reduces C3b deposition. The low deposition of C3b on OMZcnm mutant cannot be explained by increased expression of *pepO*; only 1.2-fold increase in *pepO* transcripts was observed in OMZcnm (data not shown). Moreover, reduced C3b binding was still observed in the OMZcnm/pepO double mutant, suggesting that deletion of *cnm* might influence on additional surface traits of OMZ175 which affect C3b deposition. Further studies to investigate potential interactions between PepO and Cnm on *S. mutans* surface might shed light on cooperative contributions of these proteins to complement evasion.

The recombinant collagen-binding domain of Cnm (rCnmA) directly binds to C1q but not to C4BP. Absence of rCnmA binding to C4BP is not consistent with the *cnm* mutant (OMZ175cnm) phenotype, which is defective in C4BP binding. A possible explanation for these results is that Cnm binding to C4BP would require the entire Cnm mature protein. Alternatively, Cnm binding to C4BP could require interaction with additional ligands present in serum. C4BP is a multimeric glycoprotein formed by chains of short consensus repeated CCP domains (Complement Control Protein domain). Particular CCP domains are involved in specific binding of C4b and C3b and/or to Factor I, all functions required for inhibition of the classical or lectin pathways [60,61]. CCP domains also bind to multiple host or microbial ligands including heparin [21,61,62]. Heparin binds to type I collagen and to other ECM components [63,64]. Thus, it is possible that impaired binding of C4BP to serum-treated OMZcnm was due to mutant defects in binding to serum components which in turn interact with C4BP, e.g., heparin. Finally, bacterial binding to particular plasma components, e.g. fibrinogen, may be also associated with complement evasion [64]. Thus, detailed studies on Cnm interactions with soluble serum components affecting complement deposition [65] are needed to understand how Cnm influences *S. mutans* susceptibility to complement immunity.

Deposition of C3b on bacterial surfaces promotes blood clearance through multiple mechanisms [49,50,66]. C3b/iC3b-bound bacteria are phagocytosed by blood phagocytes expressing C3b/iC3b receptors and/or removed by erythrocytes through the immune adherence process [49,^50^]. C3b-downstream functions further include bacterial lysis due to the assembly of membrane attack complex (MAC), as well as generation of extracellular and intracellular signals [49,50,66], which activate innate and adaptive immune functions in multiple host cells. Consistent with C3b-binding phenotypes, both *pepO* mutants (UApepO and OMZpepO) showed reduced survival (albeit slight) in human blood *ex vivo*, whereas inactivation of *cnm* alone did not affect bacterial survival in blood. These findings are compatible with interaction profiles of rPepO and rCnmA with serum/ECM host glycoproteins. Cnm mediates binding to host ECM proteins, specially type I collagen. On the other hand, PepO does not bind to collagen but to C4BP and plasminogen, another complement inhibitor [67], as well as to additional glycoproteins present in plasma (fibrinogen and fibronectin). The reduced capacity of the double mutant OMZ157cnm/pepO to persist in human blood compared to the OMZpepO further suggests synergistic functions of Cnm and PepO in host persistence.

Different from Cnm, PepO played a moderate role in the ability of *S. mutans* to invade HCAEC in the absence of serum components. Comprising the interface between blood and underlying tissues, endothelial cells interact with plasma and ECM glycoproteins, including those which significantly bind to PepO (plasminogen, fibrinogen, fibronectin, and laminin). Thus, PepO may facilitate host cell invasion by anchoring *S. mutans* to these glycoproteins, e.g. fibronectin, and therefore facilitating bacterial/host cell contact. PepO-mediated invasion of *S. suis* to brain microvascular endothelial cells is dependent of fibronectin [30]. Importantly, as revealed in the *G. mellonella* infection model, *pepO* gene inactivation or PepO blockage with specific antibodies clearly impacts the *S. mutans* virulence *in vivo*, in both Cnm^−^ and Cnm^+^ strain backgrounds, strengthening the importance of PepO in *S. mutans* fitness during infection.

In summary, here we showed that PepO is regulated by both VicRK and CovR, and required for immune evasion and host persistence functions. We showed that PepO interacts with multiple plasma components involved in complement down-regulation and/or host persistence, increasing resistance to C3b deposition, survival in human blood, and virulence *in vivo*. Furthermore, we provided circumstantial evidence that PepO and Cnm contribute synergistically to virulence by playing both common and unique roles in *S. mutans* ability to evade immune surveillance.
